# Delivering Mental Health Care Virtually During the COVID-19 Pandemic: Qualitative Evaluation of Provider Experiences in a Scaled Context

**DOI:** 10.2196/30280

**Published:** 2021-09-21

**Authors:** Suman Budhwani, Jamie Keiko Fujioka, Cherry Chu, Hayley Baranek, Laura Pus, Lori Wasserman, Simone Vigod, Danielle Martin, Payal Agarwal, Geetha Mukerji

**Affiliations:** 1 Women's College Hospital Institute for Health System Solutions & Virtual Care Toronto, ON Canada; 2 Women’s College Hospital Toronto, ON Canada; 3 Institute of Health System Policy, Management & Evaluation University of Toronto Toronto, ON Canada; 4 Temerty Faculty of Medicine University of Toronto Toronto, ON Canada

**Keywords:** virtual care, mental health, quality of care, implementation, COVID-19, digital health, pandemic, ambulatory care

## Abstract

**Background:**

Virtual care delivery within mental health has increased rapidly during the COVID-19 pandemic. Understanding facilitators and challenges to adoption and perceptions of the quality of virtual care when delivered at scale can inform service planning postpandemic.

**Objective:**

We sought to understand consistent facilitators and persistent challenges to adoption of virtual care and perceived impact on quality of care in an initial pilot phase prior to the pandemic and then during scaled use during the pandemic in the mental health department of an ambulatory care hospital.

**Methods:**

This study took place at Women’s College Hospital, an academic ambulatory hospital located in Toronto, Canada. We utilized a multimethods approach to collect quantitative data through aggregate utilization data of phone, video, and in-person visits prior to and during COVID-19 lockdown measures and through a provider experience survey administered to mental health providers (n=30). Qualitative data were collected through open-ended questions on provider experience surveys, focus groups (n=4) with mental health providers, and interviews with clinical administrative and implementation hospital staff (n=3).

**Results:**

Utilization data demonstrated slower uptake of video visits at launch and prior to COVID-19 lockdown measures in Ontario (pre-March 2020) and subsequent increased uptake of phone and video visits during COVID-19 lockdown measures (post-March 2020). Mental health providers and clinic staff highlighted barriers and facilitators to adoption of virtual care at the operational, behavioral, cultural, and system/policy levels such as required changes in workflows and scheduling, increased provider effort, provider and staff acceptance, and billing codes for physician providers. Much of the described provider experiences focused on perceived impact on quality of mental health care delivery, including perceptions on providing appropriate and patient-centered care, virtual care effectiveness, and equitable access to care for patients.

**Conclusions:**

Continued efforts to enhance suggested facilitators, reduce persistent challenges, and address provider concerns about care quality based on these findings can enable a hybrid model of patient-centered and appropriate care to emerge in the future, with options for in-person, video, and phone visits being used to meet patient and clinical needs as required.

## Introduction

During the COVID-19 pandemic, virtual care globally has become a foremost mechanism of health care delivery to maintain physical distancing measures, reduce personal protective equipment use, allow for redeployment of workers to COVID-19 programming, and meet public health recommendations [1-4]. Defined as “any interaction between patients and/or members of their circle of care, occurring remotely, using any forms of communication or information technologies, with the aim of facilitating or maximizing the quality and effectiveness of patient care” [5,6], virtual care has seen rapid uptake in many countries including Canada, Australia, and the United States [7-9]. This includes the province of Ontario, particularly for people in need of mental health care [10,11].

While virtual care was being offered in some settings prior to the COVID-19 pandemic as a potentially convenient and effective option for care delivery [12,13], widespread adoption was limited. Often-cited barriers to widespread implementation of video and other technology-supported virtual care include lack of digital and health literacy among providers and patients, resistance to change, and perceptions of impersonal care [13,14]. These barriers have interplayed with organizational and health policy–level factors, such as cost, the absence of mechanisms for provider reimbursement in fee-for-service systems, and concerns about regulatory constraints, leading to slower uptake and adoption of virtual care delivery, despite potential benefits [13,14]. Similar opportunities from and barriers to adoption have also been noted in mental health contexts [15-18].

In 2019, Women’s College Hospital, an academic ambulatory hospital in Toronto, Ontario, Canada, launched an institutional strategic initiative to systematically address common barriers to virtual care adoption and facilitate coordinated and widespread use of virtual care across the organization [19]. The strategy was launched with the implementation of a pilot of electronic medical record–integrated video visits within the mental health department in December 2019, prior to the onset of the COVID-19 pandemic. This department was selected for the pilot due to engaged leaders, alignment with the broader hospital strategy, provider support following a stakeholder analysis conducted by strategy implementers, and existing evidence of effectiveness and potential to increase access to mental health services [15-18,20]. The pilot included the ability for physician providers to bill for video visits on par with billing for in-person visits.

In response to the COVID-19 pandemic, the Ontario provincial government introduced physical distancing, lockdown, and other public health measures [21]. Provincial physician billing codes for phone and video virtual visits were rapidly introduced into the health care system to support access to care during lockdown measures and were available for use to providers in this study [22]. Similar to trends noted globally, scaled use of virtual care (video and phone visits) was observed in the mental health department of Women’s College Hospital during this time period.

Literature from other jurisdictions delivering mental health care virtually during COVID-19 speaks to facilitators, challenges, and perceptions of the impact of care quality [23-28]. Understanding these facilitators and challenges in a Canadian context where virtual care implementation was a strategic hospital priority can provide insights to support the use of virtual care following the reduction of physical distancing and other public health measures. It also provides an opportunity to understand consistent facilitators and persistent challenges that can occur in a scaled context despite an organizational approach to removing known barriers, as well as describe alignment with the Quadruple Aim (eg, improving patient and caregiver experience, improving population health, reducing cost, and improving the provider work environment) by looking at health service utilization and provider experience data [29]. As such, the objectives of this evaluation were to describe phone and video visit utilization and provider and staff experiences during the initial pilot phase launch of video visits in the mental health department prior to COVID-19 as well as during larger scale implementation throughout COVID-19. In particular, we sought to understand consistent facilitators of and persistent challenges to use of virtual care, as well as perceived impact on quality of care.

## Methods

### Study Design

We used a multimethods approach to collect quantitative and qualitative data for this evaluation. Quantitative data were collected through aggregate utilization data of virtual (phone and video) and in-person visits prior to and during COVID-19 lockdown measures between December 2019 and June 2020, as well as through provider experience surveys with mental health providers. Qualitative data were collected through open-ended questions on provider experience surveys, focus groups with providers in the mental health department, and interviews with administrative and implementation hospital staff. Ethics approval was received from the Research Ethics Board at Women’s College Hospital under the Ethics Assessment Process for Quality Improvement Projects (approval #: 2019-0191-E).

### Study Setting

This study took place at Women’s College Hospital, an academic ambulatory hospital located in Toronto, Ontario. Toronto is an urban city center with a diverse population of approximately 6 million people [30]. Residents of Toronto are eligible to receive provincially and publicly funded health care [31]. The study was conducted as part of Women’s Virtual, a hospital-wide strategy to implement and enable widespread adoption of virtual and digital health technologies. In December 2019, the strategy was piloted in the mental health department. Video visits were delivered by mental health providers through Zoom videoconferencing technology [32] through the electronic medical record EPIC patient portal, MyHealthRecord [33]. Self-registration by patients on MyHealthRecord was required to use and launch video visits with providers. The pilot itself was planned and supported by a team of stakeholders from across the hospital (eg., the information technology [IT], legal, and privacy staff teams), as well as within the mental health department itself including clinic managers and physician leads. Following March 2020, billing codes for phone, in addition to existing billing codes for video visits, were also available for physician providers.

### Recruitment and Data Collection

Data collection began alongside the implementation of the video visit pilot in December 2019. A purposeful sampling approach was used to email invitations to all providers in the mental health department to a focus group to discuss their experiences with video visits within the pilot project and identify opportunities for improvement. Two in-person focus groups were held in January 2020 (pre-COVID-19 pandemic), and provider consent was collected.

Following the COVID-19 pandemic and associated lockdown measures in March 2020, 2 additional focus groups were held with providers in June 2020 after widespread uptake of virtual care that included both phone and video visits across the organization. All providers in the mental health department were purposefully recruited via email with the assistance of a clinical champion in the department. After obtaining consent, focus group participants were arranged into 2 groups, 1 with only physician participants and the other with nonphysician mental health providers (such as social workers and psychotherapists), to enable tailoring of questions based on professional roles. Additional interviews were conducted with administrative staff in the mental health clinic and with staff responsible for overall virtual care implementation at the hospital. These individuals were recruited through purposeful and snowball sampling. All collected qualitative data were audio-recorded and transcribed.

Alongside focus groups and interviews, a provider experience survey was launched in May 2020 (during the COVID-19 pandemic) at the organizational level to collect data on experiences with virtual care in a scaled context, including viewpoints of providers in the mental health department. The survey included closed and open-ended questions. Aggregate utilization data on the number virtual phone, video, and in-person visits from December 2019 (3 months prior to COVID-19 lockdown measures) to June 2020 (3 months post-COVID-19 lockdown measures) was also collected for the mental health department, as well as the overall hospital.

### Data Analysis

General descriptive analysis was conducted on aggregate utilization data to understand virtual care utilization trends 3 months prior to and 3 months following the implementation of COVID-19 lockdown measures in March 2020. Similarly, quantitative data collected from surveys were analyzed using Stata SE Version 15 [34]. Data collected from open-ended questions on surveys, focus groups, and interviews were analyzed using a qualitative descriptive approach [35,36] to identify common themes within the data. Two coders (SB, JF) independently coded all survey data, and focus group and interview transcripts in NVivo [37] (qualitative data analysis software) and met to discuss codes and discrepancies. A codebook was inductively established through discussion and refined through deductive referencing of the nonadoption, abandonment, and challenges to scale-up, spread, and sustainability (NASSS) framework [38] and the Institute of Medicine’s 6 domains of health care quality framework [39]. Through the codebook, prominent and recurring themes, subthemes. and relationships were identified, discussed, and refined by SB, JF, PA, and GM. Themes and subthemes were also used to explain trends observed in utilization and survey data.

## Results

### Adoption of Video and Phone Visits

**Figure 1:**
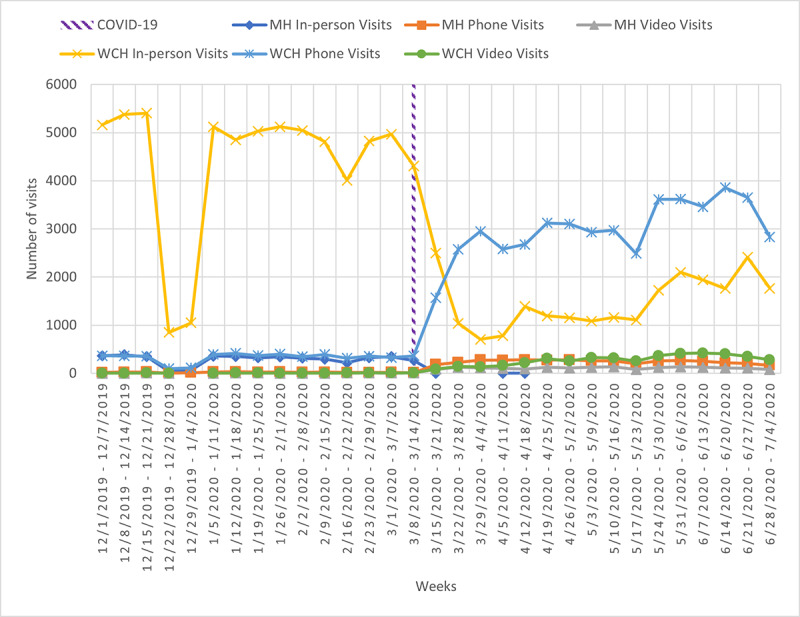
Utilization of virtual and in-person visits in the mental health (MH) department and at Women’s College Hospital (WCH) between December 2019 and June 2020.

Adoption of both phone and video visits rapidly increased during the week of March 9, 2020 to March 15, 2020, which coincides with the announcement of COVID-19 lockdown measures and availability of new provincial physician billing codes for both modalities (Figure 1). Prior to these dates, utilization of phone and video visits in the mental health department during the pilot phase (December to March) had remained low in comparison to in-person visits; physician renumeration was only available for video visits. Following the week of April 6, 2020 to April 12, 2020, phone visits began to outpace in-person visits, and the previous trend reversed. For video visits, this trend reversal for in-person versus video visits occurred during the week of May 25, 2020 to May 31, 2020. Total mental health visits showed a decreasing trend overall during this time period, likely an artefact of public health lockdown measures in effect during the same time period.

A total of 7 mental health providers participated in the pre-COVID focus groups, and a total of 14 providers participated in the focus groups held during COVID-19 (out of a total of 44 mental health providers in the department, not including fellows, residents, and students). Providers consisted of physicians, nurses, and social workers (also psychotherapists). A total of 3 administrative and implementation staff was interviewed. Of a total 34 providers from the mental health department, 30 providers (including physicians, psychotherapists, social workers) completed the provider experience survey (response rate of 88%). Table 1 provides the baseline characteristics of the provider respondents. Key themes from qualitative data and survey data describing the experiences of providers and administrative and implementation staff are subsequently presented.

**Table 1:** Baseline characteristics of the total sample for the survey (n=30).

Characteristic		n (%)
**Provider type**		
	Nurse	1 (3)
	Occupational therapist	1 (3)
	Physician	16 (53)
	Psychologist	1 (3)
	Psychotherapist	6 (20)
	Social service worker	1 (3)
	Social worker	4 (13)
**Years in practice**		
	1-2	5 (17)
	3-5	3 (10)
	6-7	5 (17)
	8-9	3 (10)
	≥10	13 (43)
	Missing	1 (3)

### Persistent Challenges for Virtual Care Use

Interview participants provided significant insights on factors influencing their adoption and use of phone and video visits during the pilot prepandemic and at scale during the pandemic (see Multimedia Appendix 1 for illustrative quotes). Key challenges to delivering virtual care are described in the following sections.

#### Operational Level: Changes in Workflows and Scheduling to Adapt to Virtual Care Delivery

Significant changes were made to adapt existing workflows to deliver care virtually. During the pilot, providers described challenges with scheduling video appointments in comparison to in-person appointments and lag times between requested and booked appointments. During COVID-19 when virtual care was adopted at scale, additional challenges were raised by administrative staff with respect to virtually checking-in patients where it was difficult to communicate when appointments were running late, thereby leading to patients waiting and wondering why the appointment had not started. Booking and checking in patients remained a persistent challenge during the pilot and at scale. Observations were also made about the increasing administrative burden with the volume of requests for appointments that were being received through the MyHealthRecord portal in comparison to prior to the onset of the COVID-19 pandemic.

#### Operational Level: Initial Setup, Troubleshooting, and Other Technology-Related Challenges in Delivering Care Virtually

Overall, 83% (25/30) of all provider survey respondents agreed or strongly agreed that the technology they used to conduct video visits was easy to use. Nevertheless, a few technical challenges were described by focus group and interview participants. One of the foremost challenges was the initial set up and MyHealthRecord registration for patients to be able to conduct video visits. Patients required education and support, especially those who had older devices or low technical literacy. This was mentioned both during the pilot phase and following scaled use during the pandemic. Other technology-related challenges were also described, including challenges during the pilot with audio, video freezing, and connectivity, which impacted the therapeutic quality of video sessions. To help with this issue, significant hospital staff were redeployed during COVID-19 to provide setup and troubleshooting support to patients. Once patients were registered on the platform and able to navigate the technology, less support was required. During the COVID-19 pandemic, rapid and scaled uptake of video visits and providers connecting to the hospital from home caused bandwidth issues, resulting in poor connectivity and lag during conducted video visits. For this reason, some providers switched to phone visits for their appointments until these issues were resolved. Phone calls continued to be an important modality to deliver care during the pandemic, as a backup when video visits were not functioning correctly, and when patients lacked access to video visit technology.

#### Behavioral Level: Increased Effort Required by Providers to Deliver Care During Transition to Virtual Care

Data from the provider survey indicated that 67% (20/30) strongly agreed or agreed that they spent the same amount of time on video visits as in-person visits during the pandemic. In contrast, only 30% (9/30) of respondents indicated that the amount of effort spent was the same as in-person visits, 43% (13/30) of respondents disagreed or strongly disagreed, and 20% (6/30) were neutral that the amount of effort spent was the same. Focus groups conducted during the pilot and during the pandemic revealed increased effort required for technology set-up and troubleshooting within video visits for themselves and patients. Providers also indicated increased administrative tasks required to conduct care virtually (eg. electronically delivering educational resources, prescriptions, and blood work requisitions to patients), which were easier to do when seeing a patient in-person. Additional effort was also required to ask for certain information (eg. vitals, weight) and help patients prepare for the appointments (eg. by inquiring if they had a private space to conduct the visit and remind them that they could not record sessions) not usually necessary during in-person visits. Lastly, providers described feelings of “Zoom fatigue” and burn out and mentioned that video visits required more concentration, energy, and adaptations to interpret visual cues in comparison to in-person visits.

### Consistent Facilitators of Virtual Care Use

Key facilitators of video visit adoption and use were also described. These are included in the following sections.

#### Operational Level: Early Targeted Pilot Prepared the Department for Virtual Care Delivery During the Pandemic

The preparation work that had occurred during the pre-existing pilot that was implemented 3 months prior to the pandemic was perceived to contribute to the rapid uptake of video visits in mental health during the COVID-19 pandemic. This included a high-touch, in-person training approach and elbow support offered to providers during the initial pilot of video visits. Similar training was also offered to patients for registration, onboarding, and troubleshooting. Since there had been significant operational planning, training, and testing in the pilot with the support of a strong clinical champion, interviewed staff felt that the department was well prepared and operationally ready to rapidly transition in-person appointments to video and phone at the onset of the pandemic. Additionally, 77% (23/30) of provider survey respondents strongly agreed or agreed that they had adequate training and resources to learn how to use video visits, and 73% (22/30) strongly agreed or agreed that they would have appropriate support if an issue were to arise with a video visit.

#### Cultural Level: Provider and Staff Acceptance and Benefits of Delivering Virtual Care

Provider survey respondents indicated that, on a scale of 1 to 10 (with 1 being not at all likely and 10 being extremely likely), they would recommend use of video visits to other providers at an average rating of 7.9 (SD 1.5; net promoter score, which determines how likely respondents are to recommend service to others, was 23.3 [40]) and phone visits at a similar average of 7.9 (SD 1.7; net promoter score of 26.7). This shows widespread acceptability among mental health providers of the use of both phone and video visits to deliver care during the pandemic. Data from focus groups and interviews suggested that this acceptability may have stemmed from the various perceived benefits of virtual visits for different stakeholder groups. For patients, provider and staff participants suggested the benefits included continuity of care during COVID-19 lockdown measures, improved access to care (such as for those with young children or living in distant locations), and improved convenience through time saved by avoiding the need to travel, take time off work, or arrange childcare both during the pilot and at scale. For providers and the organization, perceived benefits suggested during the pandemic included fewer providers working in the office, leading to freed up resources, such as clinic space, and the ability to hire more providers as a result. Lastly, several participants mentioned that there were fewer appointment cancellations during the pandemic due to patients having the added option and convenience of being able to receive care virtually.

#### System or Policy Level: Availability of Virtual Care Billing Codes for Physician Providers

Physician providers expressed the value-add of having billing codes and available financial compensation for the delivery of video and phone visits, as phone visit billing codes were unavailable to providers prior to the COVID-19 pandemic. Video and phone visits were thought to be effective, and phone visits were particularly deemed valuable to deliver certain types of visits, such as follow-up assessments, more efficiently, as well as being used as a backup modality when video technology had issues during visits or when video visit technology was not accessible or feasible for use by patients. Having billing codes for both video and phone visits enabled providers to use these modalities flexibly in the delivery of care that enabled better tailoring to patient needs.

### Perceptions on Impact on Quality Care

Overall, 43% (13/30) of the mental health providers who completed the survey strongly agreed or agreed that they felt they could deliver the same quality of care using video visits as in person, and 43% (13/30) strongly agreed or agreed the same for phone visits. Mental health providers further described their perceptions of the impact virtual care had on quality of care both during the pilot and during the pandemic, which is described in the subsequent sections under the Institute of Medicine domains of quality care [39].

#### Perceptions on Providing Appropriate and Patient-Centered Care

Mental health providers had a wide range of perspectives on the choice of appropriate virtual care modality (phone or video) for different types of visits (initial or follow-up visits) that emerged during COVID-19 where both phone and video visit modalities were being utilized. In most cases, providers felt that quality of care during phone and video visits was inferior to that of in-person care. While providers felt phone and video visits were appropriate to maintain care during the pandemic, many preferred in-person care. Choice of virtual care modality was primarily driven by patient-centered decision making, based on patient preferences, availability and accessibility of technology, and clinical appropriateness. For example, providers described the suitability of providing virtual care to patients who had difficulty leaving their homes but shared that attending in-person appointments may have helped these patients clinically and did not know what the long-term clinical impact of not leaving home for these patients might be.

#### Perceptions on the Effectiveness of Virtual Care

Virtual care was considered to be sufficient, but not excellent, quality of care in comparison to in-person care both during the pilot and during the pandemic. It was considered necessary given the pandemic context, but also valuable for certain patients and circumstances (eg. patients who would have had to travel long distances to visit the hospital or patients who have comorbidities that affect their mobility and ability to attend in-person appointments). Many providers felt that a connection with the patient was harder to build virtually, especially for new patients, which had a direct impact on the therapeutic relationship. Additionally, providers suggested that conducting visits virtually did not allow them to conduct assessment of certain visual cues that would begin in the waiting room, such as patients’ body language, mannerisms, and anxiety levels. This was expressed more in the context of phone visits, but also for video visits, thereby bringing up provider concerns related to care quality. With scaled use, providers adapted how they delivered care through phone or video to promote effectiveness. For example, video visits provided the added advantage of assessing home environments and interaction with family members, while more follow-up questions were used over the phone to understand silences. Overall, only 27% (8/30) of mental health provider survey respondents strongly agreed or agreed that the quality virtually was similar to an in-person exam, while 80% (24/30) strongly agreed or agreed that their last video visit enabled them to sufficiently address the patient’s clinical need.

#### Perceptions on Equitable Access to Virtual Care

Many providers expressed concerns regarding the accessibility of virtual care for certain patients both during the pilot and during scaled use in the pandemic. Patients’ inability to access technology or an internet connection or patients having the digital literacy to conduct virtual visits were highlighted. Moreover, patients with specific characteristics (ie, past trauma history, older age, not speaking English as a first language) were described as having more difficulties accessing and navigating the video visit technology and registration processes. In addition, some providers reported that patients had challenges accessing a private space where they could feel comfortable openly discussing their mental health concerns. Hospital staff described potential future strategies to ensure equitable access to virtual care, such as by offering devices or having private spaces with internet connection being made available at the hospital.

## Discussion

### Principal Findings

This evaluation provides insights into provider and staff experiences with mental health virtual care delivery within a pilot phase prepandemic and in a more scaled phase of implementation during COVID-19. Utilization data demonstrated the slower uptake of virtual visits in the mental health department prior to COVID-19 lockdown measures in Ontario (pre-March 2020) and increased uptake of phone and video visits during COVID-19 lockdown measures (post-March 2020). Mental health providers and clinic staff highlighted persistent barriers to use at the operational and behavioral levels including required changes in workflows and scheduling, initial set-up, troubleshooting and other technology-related challenges, and increased provider effort. Facilitators at the operational, cultural, and system/policy levels included having pre-existing infrastructure and IT support to enable widespread uptake, physician billing codes, and provider and staff acceptance of virtual care. Much of the described provider experiences focused on perceived impact on quality of mental health care delivery, including perceptions on providing appropriate and patient-centered care, perceptions on virtual care effectiveness, and equitable access to care for patients.

Results from this evaluation provide insights on alignment with the Quadruple Aim objectives, specifically on access to health care services and provider experiences. Results provide insights on provider experiences, particularly persistent barriers that can be improved to enhance use of virtual care when it is the best modality for the patient and as a complement to in-person care. For example, while 84% of all provider survey respondents agreed or strongly agreed that the technology they used to conduct video visits was easy to use, this contrasted with providers’ shared perspectives that they had ongoing challenges with technology, adapting virtual care into their clinical workflows, and the overall effort required. This might be because the technology itself was not difficult to use, but rather its integration within established clinical workflows was contingent on other individual, organizational, and policy-level factors to enable optimal efficiency [41,42]. Collective consideration of the tool (technology), the team (providers and clinical staff), and the routine (clinical workflows) may enhance positive experiences [43]. Moreover, staff suggested that the pilot initiative in the mental health department prior to COVID-19 primed providers for scaled use during the pandemic. This may be due to the considerable support provided for implementation, training, and workflow adaptation to providers and clinic staff during the pilot. This support may have ultimately shortened the learning curve and increased virtual care skill development for providers, thereby reducing provider discomfort and inexperience as a barrier to use [26]. However, despite these supports and extended periods of use, persistent challenges remained when virtual care was scaled, likely attributable to the initial transition to virtual care.

### Strengths and Limitations of the Study

The strengths of this study are two-fold. First, we examined the experiences of mental health providers who had already been delivering care virtually prior to the onset of the pandemic, as well as during scaled hospital use of virtual care. This provided an opportunity to understand the experiences and perceptions of a group of providers who had extended training and exposure to virtual care delivery, enabling us to understand consistent facilitators and persistent challenges that remained despite an organizational approach to removing known barriers. Second, we used multiple methods to examine provider experiences and use of virtual care, including focus groups, surveys, and aggregate utilization data. We also collected data from implementation and administrative staff to gather overall perspectives on virtual care implementation and adoption. Lastly, this study provides insights into provider and staff experiences within a pilot and larger scale adoption of virtual care in mental health, providing initial evidence on barriers, facilitators, and provider and staff experiences with scaled use, filling a gap in the literature where reporting on evidence in pilots is the norm [41]. Limitations in our study lie in having slightly different evaluation objectives in the pilot phase in comparison to more full-scaled implementation due to changes in scale and scope as a direct result of the unexpected COVID-19 pandemic. This reduced our capacity to compare and contrast provider experiences directly, though it provided an opportunity to describe provider experiences when virtual care was being used for a longer period of time. In addition, to avoid ethical concerns related to patient privacy, we did not include questions that could lead to discussion of personal health information such as specific patient diagnosis, and as such, this information was not collected and explored in this evaluation. Future studies can explore barriers and facilitators that could be dependent on specific patient diagnoses. Moreover, we were unable to incorporate patient perspectives in this evaluation due to feasibility constraints but will be gathering this in future phases of the evaluation. While this evaluation presents results and provider and staff experiences 3 months after the announcement of the first COVID-19 lockdown, we expect that utilization and experiences may be different in the present time, 1 year after the initial COVID-19 lockdown. This might be due to continued use of virtual care and ongoing provider learning on how to deliver care by phone and video, as well as continuous improvements in workflows and technology processes to reduce experienced challenges.

### Comparison With Prior Work

An ongoing concern among providers in this evaluation was the ability to establish therapeutic relationships during phone and video visits and impact on the quality of care, which contrasts with some studies establishing the effectiveness of mental health delivery through virtual care [44-47]. However, other studies examining provider experiences with delivering mental health care virtually have reported similar findings including the inability to fully assess nonverbal cues and potential for compromised patient privacy [23]. These constraints may hinder the therapeutic quality of virtual visits resulting in a preference to return to in-person care when it was safe to do so [23]. Some studies have also identified clinician concerns on building therapeutic relationships and challenges with technical issues [18,25,27,28,44]. Studies have suggested that the development of a specific skill set (“webside manner” [48]) may be essential to delivering care virtually, requiring provider knowledge, adaptation, practice, and eventual comfort and confidence before the use of virtual care can scale [26,48]. This can include increasing provider knowledge on virtual care effectiveness and providing safe environments for providers to establish competence and confidence in delivering virtual care [18]. Provision of high levels of support and training during the initial learning phase may additionally help to shorten providers’ learning curve, alongside guidelines on how best to triage between virtual care and in-person care modalities. We anticipate that with these supports, a hybrid model of patient-centered and appropriate care will emerge in the future, with options for in-person, video, and phone visits being used to meet patient and clinical needs as required [45,48].

### Conclusions

In conclusion, this evaluation provides insights on provider and staff experiences with virtual care use prior to and during the COVID-19 pandemic, highlighting persistent barriers, consistent facilitators, and perceived impact to quality care delivery within mental health. We have elucidated challenges to virtual care adoption in other contexts such as changes in workflows, provider adaptations on how care was delivered, technology-related issues, and provider perceptions of quality of care. This work is being used locally as a basis to develop strategies to overcome these challenges and will likely be of use in other contexts. Initiatives underway locally include support provided for virtual care implementation, training, skill, knowledge and literacy development, and workflow adaptation. Future research can continue to explore the effectiveness of mental health virtual care delivery and explore strategies that can enhance quality care delivery including gaining an understanding of patient perspectives to complement this work. Future work of this group will focus on the therapeutic relationship and equity considerations in the use of virtual care that would be beneficial, especially incorporating patient and family caregiver perspectives to further understand facilitators, challenges, and perceived and actual impact on the quality of virtual care.

## Appendix

Multimedia Appendix 1 Supplementary Appendix 1: Themes and illustrative quotes.

## Abbreviations

IT: information technology
NASSS: non-adoption, abandonment, scale-up, spread, sustainability

## References

[1] Webster P (2020). Virtual health care in the era of COVID-19. Lancet.

[2] Ahmed S, Sanghvi K, Yeo D (2020). Telemedicine takes centre stage during COVID-19 pandemic. BMJ Innov.

[3] Important COVID-19 Information and Updates. Ontario Telemedicine Network.

[4] Heyworth L, Kirsh S, Zulman D, Ferguson JM, Kizer KW (2020). Expanding Access through Virtual Care: The VA’s Early Experience with Covid-19. New England Journal of Medicine Catalyst.

[5] Jamieson T, Wallace R, Armstrong K, Agarwal P, Griffin B, Wong I (2015). Virtual Care: A Framework for a Patient-Centric System. Women’s College Hospital Institute for Health Systems Solutions and Virtual Care.

[6] Shaw J, Jamieson T, Agarwal P, Griffin B, Wong I, Bhatia RS (2018). Virtual care policy recommendations for patient-centred primary care: findings of a consensus policy dialogue using a nominal group technique. J Telemed Telecare.

[7] Snoswell CL, Caffery LJ, Haydon HM, Thomas EE, Smith AC (2020). Telehealth uptake in general practice as a result of the coronavirus (COVID-19) pandemic. Aust Health Rev.

[8] Mehrotra A, Chernew M, Linetsky D, Hatch H, Cutler D, Schneider EC (2021). The Impact of COVID-19 on Outpatient Visits in 2020: Visits Remained Stable, Despite a Late Surge in Cases. The Commonwealth Fund.

[9] Robinson J, Borgo L, Fennell K, Funahashi TT (2020). Covid-19 Pandemic Accelerates the Transition to Virtual Care. New England Journal of Medicine Catalyst.

[10] Bhatia RS, Chu C, Pang A, Tadrous M, Stamenova V, Cram P (2021). Virtual care use before and during the COVID-19 pandemic: a repeated cross-sectional study. CMAJ Open.

[11] Glazier RH, Green ME, Wu FC, Frymire E, Kopp A, Kiran T (2021). Shifts in office and virtual primary care during the early COVID-19 pandemic in Ontario, Canada. CMAJ.

[12] Kruse CS, Krowski N, Rodriguez B, Tran L, Vela J, Brooks M (2017). Telehealth and patient satisfaction: a systematic review and narrative analysis. BMJ Open.

[13] Fisk M, Livingstone A, Pit SW (2020). Telehealth in the Context of COVID-19: Changing Perspectives in Australia, the United Kingdom, and the United States. J Med Internet Res.

[14] Scott Kruse C, Karem P, Shifflett K, Vegi L, Ravi K, Brooks M (2018). Evaluating barriers to adopting telemedicine worldwide: A systematic review. J Telemed Telecare.

[15] Hensel J, Shaw J, Ivers N, Desveaux L, Vigod S, Cohen A, Onabajo N, Agarwal P, Mukerji G, Yang R, Nguyen M, Bouck Z, Wong I, Jeffs L, Jamieson T, Bhatia RS (2019). A Web-Based Mental Health Platform for Individuals Seeking Specialized Mental Health Care Services: Multicenter Pragmatic Randomized Controlled Trial. J Med Internet Res.

[16] Hoffman L, Benedetto E, Huang H, Grossman E, Kaluma D, Mann Z, Torous J (2019). Augmenting Mental Health in Primary Care: A 1-Year Study of Deploying Smartphone Apps in a Multi-site Primary Care/Behavioral Health Integration Program. Front Psychiatry.

[17] Mahmoud H, Vogt EL, Sers M, Fattal O, Ballout S (2019). Overcoming Barriers to Larger-Scale Adoption of Telepsychiatry. Psychiatric Annals.

[18] McClellan MJ, Florell D, Palmer J, Kidder C (2020). Clinician telehealth attitudes in a rural community mental health center setting. Journal of Rural Mental Health.

[19] Women's Virtual. Women's College Hospital.

[20] Stiens L (2019). Uses, Benefits, and Future Directions of Telepsychiatry. Iowa State University.

[21] (2020). Ontario Enacts Declaration of Emergency to Protect the Public. Office of the Premier.

[22] Keeping Health Care Providers informed of payment, policy or program changes. Ontario Ministry of Health.

[23] Uscher-Pines L, Sousa J, Raja P, Mehrotra A, Barnett ML, Huskamp HA (2020). Suddenly Becoming a "Virtual Doctor": Experiences of Psychiatrists Transitioning to Telemedicine During the COVID-19 Pandemic. Psychiatr Serv.

[24] Sasangohar F, Bradshaw MR, Carlson MM, Flack JN, Fowler JC, Freeland D, Head J, Marder K, Orme W, Weinstein B, Kolman JM, Kash B, Madan A (2020). Adapting an Outpatient Psychiatric Clinic to Telehealth During the COVID-19 Pandemic: A Practice Perspective. J Med Internet Res.

[25] Rosic T, Lubert S, Samaan Z (2020). Virtual psychiatric care fast-tracked: reflections inspired by the COVID-19 pandemic. BJPsych Bull.

[26] Rosen C, Glassman L, Morland L (2020). Telepsychotherapy during a pandemic: A traumatic stress perspective. Journal of Psychotherapy Integration.

[27] Reay RE, Looi JC, Keightley P (2020). Telehealth mental health services during COVID-19: summary of evidence and clinical practice. Australas Psychiatry.

[28] Connolly SL, Stolzmann KL, Heyworth L, Weaver KR, Bauer MS, Miller CJ (2021). Rapid Increase in Telemental Health Within the Department of Veterans Affairs During the COVID-19 Pandemic. Telemed J E Health.

[29] Bodenheimer T, Sinsky C (2014). From triple to quadruple aim: care of the patient requires care of the provider. Ann Fam Med.

[30] (2016). Census Profile, 2016 Census. Statistics Canada.

[31] Apply for OHIP and get a health card. Government of Ontario.

[32] Zoom.

[33] MyChart. EPIC.

[34] StataCorp LLC.

[35] Sandelowski M (2010). What's in a name? Qualitative description revisited. Res Nurs Health.

[36] Sandelowski M (2000). Whatever happened to qualitative description?. Res Nurs Health.

[37] NVivo.

[38] Greenhalgh T, Wherton J, Papoutsi C, Lynch J, Hughes G, A'Court C, Hinder S, Fahy N, Procter R, Shaw S (2017). Beyond Adoption: A New Framework for Theorizing and Evaluating Nonadoption, Abandonment, and Challenges to the Scale-Up, Spread, and Sustainability of Health and Care Technologies. J Med Internet Res.

[39] Institute of Medicine (US) Committee on Quality of Health Care in America (2001). Crossing the Quality Chasm: A New Health System for the 21st Century.

[40] Reichheld FF (2003). The One Number You Need to Grow. Harvard Business Review.

[41] Lokken TG, Blegen RN, Hoff MD, Demaerschalk BM (2020). Overview for Implementation of Telemedicine Services in a Large Integrated Multispecialty Health Care System. Telemed J E Health.

[42] Desruisseaux M, Stamenova V, Bhatia RS, Bhattacharyya O (2020). Channel management in virtual care. NPJ Digit Med.

[43] Shaw J, Agarwal P, Desveaux L, Palma DC, Stamenova V, Jamieson T, Yang R, Bhatia RS, Bhattacharyya O (2018). Beyond "implementation": digital health innovation and service design. NPJ Digit Med.

[44] Hubley S, Lynch SB, Schneck C, Thomas M, Shore J (2016). Review of key telepsychiatry outcomes. World J Psychiatry.

[45] Chakrabarti S (2015). Usefulness of telepsychiatry: A critical evaluation of videoconferencing-based approaches. World J Psychiatry.

[46] Bischoff R, Hollist C, Smith C, Flack P (2004). Addressing the Mental Health Needs of the Rural Underserved: Findings from a Multiple Case Study of a Behavioral Telehealth Project. Contemporary Family Therapy.

[47] Flodgren G, Rachas A, Farmer AJ, Inzitari M, Shepperd S (2015). Interactive telemedicine: effects on professional practice and health care outcomes. Cochrane Database Syst Rev.

[48] Smith K, Ostinelli E, Macdonald O, Cipriani A (2020). COVID-19 and Telepsychiatry: Development of Evidence-Based Guidance for Clinicians. JMIR Ment Health.

