# Supplementary material for: Delivering Mental Health Care Virtually During the COVID-19 Pandemic: Qualitative Evaluation of Provider Experiences in a Scaled Context
Source: JMIR Form Res. 2021 Sep 21;5(9):e30280. doi: 10.2196/30280 (PMC8457338; doi:10.2196/30280)
Supplement: Multimedia Appendix 1 [file formative_v5i9e30280_app1.docx]

| *Theme & Subtheme* | *Quotes* |
| --- | --- |
| Persistent Challenges for Virtual Care Use - Operational Level: Changes in Workflows and Scheduling to Adapt to Virtual Care Delivery | “And so, then that just becomes even more complicated when it comes to virtual visits because the patient is not there, just walking to reception to book their follow-up.” – Mental health provider during pilot pre COVID-19  “Before it wasn’t like not a lot of patients are using this system because they are not aware of much of this that they can sign up to MyChart and then they communicate….Before the pandemic we have only average numbers of requests. But during this time, I can say it’s really they are more comfortable using this way of communication and request through appointments.” – Hospital staff during COVID-19 |
| Persistent Challenges for Virtual Care Use *-* Operational Level: Initial Setup, Troubleshooting, and Other Technology-Related Challenges in Delivering Care Virtually | “Some patients, especially in the beginning, the first couple of weeks, they would have technical challenges or not many people were signed up for myHealthRecord. So, when we were calling people to say, okay, your visit now is going to be virtual, because of the COVID situation and you must sign up for myHealthRecord, some of them would say, yeah, sure, I’ll sign up for it, but then they would forget or they chose not to do it. And then it was time for their appointment and of course they couldn’t launch the video visit without being signed up and then there was a delay and they missed the ten-minute window of being able to check in and then you’d have to make a new appointment, basically.” – Hospital staff during COVID-19  “In my experience the clients all preferred video, but working from home the bandwidth, not my internet connection but the working within the….network, did not support video. There was a lot of lag, so we had to be creative. We just switched to video with some people. Some people we kept the video on, muted it, and then I called the person to have the audio, so we still had the face to face connection…otherwise, everyone seemed to have preferred video, and I have too. But technology has limited whether that was available or not.” – Mental health provider during COVID-19 |
| Persistent Challenges for Virtual Care Use *-* Behavioral Level: Increased Effort Required by Providers to Deliver Care During Transition to Virtual Care | “I keep saying I’m so tired, I’m so tired, I’m so tired, and I don’t know why, but I’m still doing back-to-back. I’m still doing back-to-back on video and phone, and at the end of the day, I’m pulling my head together trying to call in the scripts, trying to do the blood work, trying to do a letter, trying to do this, and trying to do that.” – Mental health provider during COVID-19  “The administrative burden is now outside. Some of the administrative things that you would have done in the appointment, like, printing a script, or giving someone a blood req, all those things are now all outside of the appointment time.” – Mental health provider during COVID-19  “I think there can be effort to sort of troubleshoot I guess. For me there was a visit where I couldn’t hear the patient and so it was just sort of playing around and figuring out my computer settings, her computer. There’s that, again, a potential delay.” – Mental health provider during COVID-19  “Video visits also take more effort because my patients and I often find it difficult to hear clearly so time is spent repeating or there may be a lag time. It also takes more effort and time when there are technical difficulties with EPIC or the audio for example.” - Mental health provider survey response during COVID-19 |
| Consistent Facilitators of Virtual Care Use *-* Operational Level: Early Targeted Pilot Prepared the Department for Virtual Care Delivery During the Pandemic | “They were pretty much ready to go because the department was primed and ready to go. Mental health was pretty much ready for video overnight, I would say, aside from just the providers having to learn how it worked.” – Hospital staff during COVID-19  “Getting provider buy-in prior to the pandemic wasn’t actually that difficult because the virtual strategy is also mandated by the hospital’s overall strategy. It was very easy for the virtual team to walk into a room and have interest right off the bat.” – Hospital staff during COVID-19 |
| Consistent Facilitators of Virtual Care Use - Cultural Level: Provider and Staff Acceptance and Benefits of Delivering Virtual Care | “Yeah, I would say it’s been really helpful, again, in terms of people that otherwise I think couldn’t get in where I thought it was important for them to come. So whether that was due to just distance or child being sick and not being able to bring them in and needed to be at home.” – Mental health provider during pilot pre COVID-19  “Largely, I think it’s convenience for patients. I think there are a lot of patients who we see, because our population is quite far-reaching and because we are quite specialized, who don’t live anywhere near the hospital. It’s one thing, I think, to come and to meet your provider, but then after that, I don’t know that patients absolutely need to come here if they don’t want to…I think it’s useful from an access perspective. I think we will be able to provide care to patients that we previously hadn’t, and provide better care to patients that we already provide care to from an access standpoint.” – Mental health provider during COVID-19  “With that population, they’re ill and they have mental health issues, so we had a high cancellation and no-show rate. For that, we found it was very helpful as well. Patients, sometimes they can’t get out of bed that day or they just feel they can’t get dressed and get here, so the video visits worked for them.” – Hospital staff during COVID-19  “If we can have providers that are only on-site 50% of the time, and therefore only taking up an office 50% of the time, that gives us room to have almost double our amount of physicians providing care.” – Hospital staff during COVID-19 |
| Consistent Facilitators of Virtual Care Use - System or Policy Level: Availability of Virtual Care Billing Codes for Physician Providers | “If I was to fantasize about COVID-19 being gone, I would like to still have the capacity, particularly for the phone actually, even more than the video, to be able to bill for that because I think there are ways that that’s really useful for certain kinds of care.” – Mental health provider during COVID-19  “To be able to get paid to have the visit by virtual, whether it’s phone or video, that makes sense, and there’s no way that that’s necessarily going to be less effective than when they came in person.” – Mental health provider during COVID-19 |
| Perceptions on Impact on Quality Care - Perceptions on providing appropriate and patient-centered care | “… it would be based on client comfort and barriers. So, if a client felt due to a number of things, convenience or financial resources or transportation or schedule felt that video would be better for them, and that was also assessed within what’s therapeutically beneficial, assessing the avoidance that might be playing out in that, like doing a more comprehensive assessment, then I would be happy to do video if it didn’t seem like it would be therapeutically disadvantaging them in some way.” – Mental health provider during COVID-19  “I’m doing the assessments for group, these 45-minute assessments, on the phone. I feel I wish I could do that for eternity. They’re just efficient and effective. People are happy to not have to come in for that. But if I’m doing therapy, then I’d rather be doing it in person. I think it’s a better connection. We’re using much more of our full selves I think in person than on video or phone so I think it depends a little bit on the context.” - Mental health provider during COVID-19 |
| Perceptions on Impact on Quality Care - Perceptions on the effectiveness of virtual care | “…I have a sense that it’s going to be harder to kind of build a therapeutic relationship with someone if you’ve only met them once and then all your visits are virtual. Again, it might matter less in other programs, like in mental health that’s pretty key. Your capacity to sense what’s going on in the person, what it feel like to sit with them, what their level of anxiety is, there are subtle things that I think you might miss if you’re only doing virtual or virtual is happening and you’re not getting people in that often.” – Mental health provider during COVID-19  “…with some of the newer people I’ve picked up and started with, there’s more of a casual feel or something. I’ve had to kind of reiterate or state what the therapeutic relationship is, and this is protected time, privacy, some of those boundaries in a way that I haven’t had to reiterate before.” – Mental health provider during COVID-19 |
| Perceptions on Impact on Quality Care - Perceptions on equitable access to virtual care | “I have had clients walking outside on their phone to get some private space. Also, the complication with our current social isolation, family has one computer and one of the partners is working on the computer so there is no access. And then the phone has to be used at that point.” – Mental health provider during COVID-19  “It’s different to say to your son or to your husband can you help me set-up for my appointment with, I don’t know, the endocrinologist to look at my thyroid than to talk to my trauma therapy therapist, right?” – Mental health provider during COVID-19 |
